# The Female Urinary Microbiota/Microbiome: Clinical and Research Implications

**DOI:** 10.5041/RMMJ.10292

**Published:** 2017-04-28

**Authors:** Linda Brubaker, Alan J. Wolfe

**Affiliations:** 1 Department of Obstetrics & Gynecology, University of California San Diego, CA, USA; 2 Department of Urology, University of California San Diego, CA, USA; 3 Department of Microbiology and Immunology, Stritch School of Medicine, Loyola University Chicago, IL, USA

**Keywords:** Female urinary disorders, urinary microbiome, urinary microbiota

## Abstract

The changing science of the urinary microbiota and microbiome has both clinical and research implications. This review paper provides an overview of the state of this science, as well as a discussion of the potential for prevention, diagnosis, and treatment of human disease. The history of techniques used for clinical detection of infection are placed into context along with the modern methods of bacterial detection and identification.

## INTRODUCTION

Human microbiome research has provided opportunities for new insights into prevention, diagnosis, and treatment of human disease. The Human Microbiome Project, a major research initiative funded by the National Institutes of Health (NIH), began the process of cataloguing the core microbial composition of healthy adults to determine how changes to microbial communities (microbiota) affect human health. To identify microbes, these projects did not rely on traditional culture techniques used clinically to identify pathogens; instead, they relied on DNA-sequencing approaches that differ from the tests that clinicians use to study human DNA. One DNA-sequencing approach, 16S ribosomal RNA (rRNA) gene sequencing, detects variable portions of a gene present in all bacteria. To identify the bacteria present in a mixed sample, such as feces or urine, the patterns (sequences) of the detected portions are matched to known sequences.

Using 16S rRNA gene and other modern sequencing techniques, researchers have determined the microbial composition of healthy skin,^1^,^2^ gastrointestinal tract,^3^–^5^ mouth,^6^–^8^ and vagina.^9^–^12^ Multiple clinical correlations have been discovered—the list grows daily. For example, investigators have documented microbiota alterations associated with neonatal necrotizing enterocolitis, cystic fibrosis, Crohn’s disease, some forms of sexually transmitted genitourinary disease, bacterial vaginosis, and pregnancy.^13^–^17^ To date, the most-studied microbiome is that of the gastrointestinal tract; researchers have observed strong linkages between the gut microbiota and common conditions such as obesity.^18^,^19^ Important treatment advances in clinical treatment have been made, such as fecal transfers for treatment of refractory or life-threatening *Clostridium difficile* gastrointestinal infection.

Although the female urinary microbiota (FUM) was not included in the original or subsequent demonstration Human Microbiome Project (HMP), there is now a growing body of evidence confirming the presence of the FUM in adult women. Urinary microbiome researchers have used 16S rRNA sequencing to detect bacterial DNA in the human female bladder of women without clinical infection, regardless of the presence or absence of other urinary tract symptoms.^20^–^23^ To obtain evidence of live microbes in urines deemed negative by traditional urine culture techniques, researchers developed enhanced culture techniques.^21^,^24^–^26^ These enhanced techniques, described in detail later in this review, confirmed that sequenced urinary bacteria are alive and confirmed the presence of a microbial community in the urinary bladders of adult women—the FUM. These reproducible independent findings change the commonly held misconception that the bladder is a sterile environment—it is not.

Studies to date reveal that the FUM is composed of a mixture of bacteria (as well as less-studied viruses and fungi), and there is solid evidence that the characteristics of the FUM appear related to certain common urinary conditions, such as urgency urinary incontinence (UUI)^20^,^21^ and urinary tract infection (UTI).^27^–^29^ This review provides context, interpretation, and perspective on our current understanding of the FUM; in addition, we highlight research challenges and frontiers. This review does not include male, pediatric, neurogenic, or obstetric populations, or include upper urinary tract conditions, e.g. urinary stones.

## OVERVIEW OF HUMAN MICROBIOME SCIENCE

The dual concepts that most human body sites are colonized with bacteria and that the majority of those bacteria are non-pathogenic are recent entries into clinical thought. Because 150 years of medical studies tended to focus on disease-causing (pathogenic) bacteria, we know very little of the human body’s natural and generally non-pathogenic residents (microbiota, often called normal flora or commensals). The microbiota are quite numerous; in the human body, there are 10 microbes for every one human cell, and the vast majority of those microbes are not pathogens. Indeed, we cannot live without them. They degrade complex carbohydrates, generate energy, synthesize vitamins, educate our immune systems, and protect us against invading pathogens.

Researchers associated with the Human Microbiome Project (HMP), a major research initiative funded by the National Institutes of Health (NIH), performed the first large-scale mapping of the human microbiota. To detect and identify individual members of the microbiota, they primarily used culture-independent, DNA-based methods (most often 16S rRNA gene sequencing) developed by microbial ecologists and tools funded by the Human Genome Project. The HMP researchers initially studied the gut microbiota because samples from this heavily colonized (high biomass) environment are easy to collect, there exists substantial diversity between individuals,^3^ and differences were previously reported in health and disease states.^30^ These researchers quickly expanded their efforts to sample the microbiota of 242 healthy, mostly young individuals, collecting samples from 18 body habitats from 5 major body areas: GI tract, mouth, vagina, skin, and nasal cavity.^31^

This seminal work provided a framework for researchers to characterize human microbiota in health and disease. Human microbial community structures are complex, and there can be significant variations between individuals and within individuals over time. To date, however, a limited number of studies have described the longitudinal communities within individuals.^32^ The continuing human microbiome research suggests the paradigm of humans as supraorganisms with complex interactions of microbial and human cellular as well as genetic components. Much remains to be learned; yet, fundamental discoveries are already informing human health.

Beyond the simple concept that microbes are present, certain community characteristics can describe the microbiota. For example, descriptors based on the most prevalent microbe may help describe a community and distinguish it from other communities. In order to better characterize microbial community attributes, human microbiome investigators have developed a qualitative approach using identification by community type; they are called enterotypes in the gut,^33^ community state types (CSTs) in the vagina,^15^ and urotypes in the urine.^21^ To determine community types, bacterial profiles are clustered together based on taxonomic similarity. Those clusters are tested for their statistical association between individuals and diseases. This method of community characterization has been used to describe the different enterotypes that are correlated to one’s diet, obesity, or Crohn’s disease.^33^–^35^

Significant advances in our understanding of the vaginal microbial community occurred using HMP data to describe community state types. While it had been previously known that the vagina is highly colonized with bacteria (high biomass), we now know that the vaginal microbiota of women of reproductive age tends to fall into one of five community state types (community groupings by dominant microbe). Only one community state type is dominated by a diverse group of anaerobes, including *Anaerococcus*, *Peptoniphilus*, *Prevotella*, *Streptococcus*, *Atopobium*, and *Gardnerella*.^15^ The remaining community state types are dominated by different species of *Lactobacillus* (*L. crispatus*, *L. gasseri*, *L. iners*, and *L. jensenii*, respectively).^15^

Another approach to description of the human microbial community uses ecological parameters, including diversity, stability, and/or resilience.^36^ Recently, Lloyd-Price and co-workers delineated the current understanding of the characteristics of “healthy” microbiota.^36^ Despite multiple challenges to the dichotomous usage of the term “healthy microbiota,” several key observations can be made. First, community characteristics often depend on attributes of more than a single microbe and commonly feature a “core” set of microbes. Second, the core functions of the “healthy” microbiota are often quite similar, despite variability in the meta-genome of the human microbiota (i.e. the genomes of all members of the microbiota). Thus, even in the absence of disease, microbiota in many areas of the human body have a large degree of interpersonal diversity.^31^

Human microbial communities respond to environmental context; this has been best studied in the human gut, where strong evidence supports the relationship between microbial variation, dietary intake, and body mass index. The clinical implications of these relationships are significant, as researchers have documented clear connections with the human brain.^37^ Given the central role of the nervous system in urinary control, the urinary microbiota likely has some forms of central nervous signaling that is yet to be discovered. These future discoveries may help unlock mysteries that have limited our insights into common urinary disorders in adult women.

## THE EVOLUTION OF URINARY TRACT BACTERIAL DETECTION

Clinicians may often empirically treat symptoms caused by suspected uropathogens. Beyond a simple symptoms history, the clinician may rely on ancillary testing, such as office-based dipstick assessment of a fresh urine sample. These office-based dipsticks do not have a role in identifying specific uropathogens; rather, these tests screen for factors known to be associated with UTI, such as leukocyte esterase and nitrates. While this clinical practice has been considered pragmatic, expediting care to patients, it has the risk of resulting in unnecessary antibiotic use. As the collateral consequences of systemic antibiotic use are better understood, clinicians and patients alike must become better stewards of antibiotics.

Since the mid-1950s, the gold standard for detection of uropathogenic microbes has been the standard clinical microbiology urine culture protocol, a method originally designed to detect patients with pyelonephritis.^38^ Unfortunately, use of the standard urine culture has been expanded well beyond this original purpose, without any empirical evidence that this generalization was valid. Although most clinicians consider the standard urine culture to be the gold standard for identifying the pathogens assumed to be causing infection in the urinary tract, including cystitis (bladder infection) and pyelonephritis (kidney infection), problems do exist. First, the common threshold for an infection diagnosis (i.e. ≥10^5^ colony-forming units per milliliter [CFU/mL] of a known uropathogen) is debated, as several studies argue that lower thresholds should be used to detect significant bladder infection in symptomatic women.^39^–^42^ More problematic is the fact that most bacteria do not culture by standard urine culture protocols, which were designed to detect common fast-growing pathogens with basic nutrient needs and no aversion to oxygen, especially *Escherichia coli*, the most common UTI cause. As the standard protocol does not detect anaerobes, slow growers, or bacteria with complex needs, most urine cultures are deemed negative, even though they do contain bacteria, as shown by DNA sequencing. Because it is very sensitive, sequencing of DNA challenges the role of the standard urine culture as a gold standard. However, researchers and clinicians alike must be extremely careful about contamination from other sources (not unlike traditional urine testing). The earliest DNA sequencing-based study of urine obtained from the female urinary bladder was carefully controlled to avoid contamination by bacteria from other sites (such as vagina, gut, or skin). To control for possible contamination of the sample by skin microbes, samples obtained by suprapubic aspiration (intraoperative) were compared with simultaneously obtained control swabs of the sample site and samples from suprapubic needles that punctured the skin, but did not enter the bladder. The suprapubic aspirated urine samples also were compared to those obtained by transurethral catheterization. This comparison revealed that urine obtained by transurethral catheter and suprapubic aspirate closely resembled each other microbiologically. In contrast, bacteria in “clean catch” mid-stream urines more closely resembled the bacteria found in vaginal swabs. This confirmed that urine taken directly from the bladder contains bacteria, as suprapubic aspiration bypasses the vagina. It also confirmed that transurethral catheterization was a proper urine collection method for study of the bladder microbiota and that voided “clean catch” was not.^23^

While it is likely that bacterial detection using point-of-care DNA-based tests will move into clinical settings in the near future, sequencing is not practical for most clinical settings at this time. Instead, improvements in urine culture techniques offer an alternative that can be implemented immediately in most clinical laboratories.

## HOW CULTURING WORKS—ADVANCES IN CULTURING TECHNIQUES: DETAILS FOR CLINICIANS

Historically, clinically relevant organisms have been classified and named using morphologic and phenotypic comparisons to type (i.e. typical) strains described in standard references, e.g. *Bergey’s Manual of Systematic Bacteriology* and the *Manual of Clinical Microbiology*,^43^,^44^ or to those found within the American Type Culture Collection (ATCC). This practice has significant limitations for characterization of the large number of microbial species that may reside in a complex microbiota.

To remind readers, the taxonomic hierarchy moves from more general to less general, with domain, kingdom, phylum—followed by class, order, and family—and more specifically genus and species. Typically, clinicians are used to microbial names that describe a bacterium by its genus and species, such as *Escherichia coli*. For this organism, the genus is *Escherichia* and the species is *coli*. Within *E. coli*, however, are multiple strains with varying characteristics. Some *E. coli* strains are pathogenic: for example, strains that are uropathogenic (UPEC), enterohemorrhagic (EHEC), or enterotoxigenic (ETEC). Many clinicians are not aware, however, that the majority of *E. coli* strains are non-pathogenic and instead are members of the “healthy” intestinal microbiota. Indeed, some *E. coli* strains are used as probiotics.^45^

Most clinical urine samples are collected from voided urine, despite the known risk of contamination from the vulva. The urine is sent to the laboratory using a tube meant to inhibit growth during specimen transport. Standard urine culture techniques involve plating of a prespecified aliquot of urine onto culture media. Although there are slight variations across clinical laboratories, a typical protocol is performed by inoculating 0.001 mL of urine onto each of two different media (5% sheep blood agar plate [BAP] and MacConkey agar) and streaking each plate surface to obtain quantitative colony counts. After incubation under aerobic conditions at 35°C for 24 h, each separate morphological colony type is counted and identified in any amount. Because the plated volume is 0.001 mL, the detection level is 10^3^ CFU/mL, represented by 1 colony of growth on either plate. When no growth is observed, the cultures are reported as “no growth” (of bacteria at lowest dilution, i.e. 1:1,000).

Many members of the FUM detected by sequencing do not grow under standard urine culture conditions. However, simple refinements to the standard urine culture protocol (increased volume, various growth conditions, increased duration of incubation) allow detection of many additional urinary microbes. For example, our research team established an improved protocol called enhanced quantitative urine culture (EQUC) that dramatically improves detection of clinically relevant urinary microbes.^25^ Others have established similar approaches.^24^,^26^ We recently recommended a streamlined version of EQUC for use in clinical microbiology laboratories that uses the following conditions: 0.1 μL urine obtained by transurethral catheter plated onto BAP, MacConkey, and colistin nalidixic acid (CNA) agars, with incubation of all agars in 5% CO^2^ for 48 h.^28^

The information from the improved urine culture techniques provides a more complete reporting of the microbes present in an individual’s urinary microbiota. However, with this additional information comes the challenge of clinical interpretation. The new paradigm acknowledges the possibility of a spectrum of organisms (“good” and “bad”) within the FUM. For example, several studies provide evidence of the presence of protective FUM members.^21^,^27^ Thus, the clinician must determine the appropriate treatment, while recognizing that the clinical goal is no longer to kill all microbes that are present.

## HOW SEQUENCING WORKS—DETAILS FOR CLINICIANS

Earlier in this paper, we provided an overview of how sequencing works. Here, we provide additional information to enhance the clinician’s understanding of this foundational technique. The most common high-throughput DNA-sequencing technologies generate relatively short sequence reads (~250–400 base pairs); however, these massively parallel sequencing approaches (often called next-generation sequencing or NGS) produce many sequences and thus yield great depth of a single sequence or extensive coverage of a genome. Two types of sequencing strategy exist: whole-genome and metagenome. Whole-genome sequencing is used to delineate the genome of a particular bacterium; thus, it starts with an isolated pure culture. In contrast, metagenomic sequencing is performed on mixed populations, such as a swab, feces, or urine, with the intent to identify the diverse bacteria present in that mixture. Each strategy uses a different method to turn the large amounts of short sequences into valuable information.

Human Microbiome Project research has generally relied on 16S rRNA sequencing to classify bacteria within a clinical sample. The 16S rRNA gene is highly conserved amongst bacteria.^46^–^48^ Within the gene, however, some portions evolve. Thus, conserved sequences are interspersed with hypervariable sequences. The latter contain specific sequence differences that are used to measure phylogenetic relatedness. There are nine of these hypervariable sequences, and together they contain sufficient genetic differences to identify most bacteria to the species level. However, the information available in a single hypervariable region suffices for assignment only to the family or genus levels. Furthermore, the method is not quantitative because it relies on amplification by the polymerase chain reaction. Since different sequences amplify at different rates, the number of sequence reads from different samples cannot be directly compared. Instead, researchers compare these data qualitatively, looking at the relative amounts of bacteria, rather than the numbers of raw sequences.

## WHAT WE KNOW NOW ABOUT THE FUM AND HOW WE LEARNED IT

Since the first publication in 2012 describing the presence of bacteria in the female urinary bladder,^23^ researchers have learned a significant amount about the FUM in health and in women affected by common urinary disorders. As expected, compared to the human gut or oral cavity, the FUM has fewer organisms and is less complex.

We have learned that the FUM is detected in nearly all, but not every, urine analyzed. Using the prior dogma of “sterility,” it is tempting to call this minority of urine “sterile”; however, even using the sensitive technique of sequencing, it is likely that certain FUM microbes are below our current limits of detection. Thus, we recommend the description of “below detection limits” rather than “sterile” to describe urine specimens that have no sequenced bacterial DNA.^49^

In the majority of urine samples that provide 16S rRNA sequences, a main feature of the FUM is its tendency to be dominated by a single genus, most often by *Lactobacillus.* Less commonly, *Gardnerella*, *Streptococcus*, *Staphylococcus*, or *Corynebacterium* may be the dominant genus*.* Much less often, the genera *Aerococcus*, *Actinomyces*, or *Bifidobacterium* dominate. Sometimes, the dominant taxon is the family Enterobactericeae, which contains the genus *Escherichia.* In a subset of women, there is no dominant genus or family. A second feature is the diversity of microbes within the community. The FUM of some women have many organisms, while others have very few or even only a single detected microbe (essentially a monoculture).^20^–^22^,^25^,^28^,^49^,^50^

The relationship with adjacent pelvic microbial niches, notably the vagina and the gut, is not well understood, although studies are underway. Researchers are working to determine whether the bladder and vagina share a common community of microbes, whether microbes move between these niches, and if some microbes favor one niche over another. *Lactobacillus* species are common dominant members of the vaginal microbiota and are considered to be protective. Studies thus far reveal that while *Lactobacillus* is present within both microbial niches, the specific species of *Lactobacillus* may vary.^9^–^12^,^20^–^22^,^25^,^28^,^50^ Moreover, there appears to be a relationship to clinical status, with *L. gasseri* being more common in the bladders of women with UUI and *L. crispatus* being more common in women without lower urinary tract symptoms.^21^ These findings suggest that the role of *Lactobacillus* may differ in the bladder compared to the vagina. As research teams sequence the genomes of strains isolated from different pelvic floor sites of the same individual, we will gain insight into these important relationships.

Little is known about the specific role of FUM organisms. Certain microbes have characteristics that protect against uropathogens by producing antibiotics, antimicrobial peptides, and/or other antimicrobial compounds that inhibit or kill other microbes. For example, certain *Lactobacillus* species that colonize the vagina excrete lactic acid and hydrogen peroxide, inhibiting the growth of uropathogenic *E. coli*.^51^ Microbes can also inhibit pathogens by outcompeting them for host receptors^52^ or scarce nutrients.^45^

The stability of the FUM requires further study. It is well known that the vaginal and gut microbiota are able to rapidly change microbial composition. Such studies have yet to be performed in the FUM. And, researchers must learn whether changes in the vagina and/or gut are accompanied by changes in the urinary microbiota.

## THE IMPORTANT QUESTIONS FOR CLINICAL CONDITIONS OF INTEREST

Increasingly, clinicians are faced with the knowledge of the collateral effects of systemic antibiotic use. The disruption to healthy microbiota is well known following a single course of systemic antibiotics; this is exacerbated in patients with multiple, repeated exposures to systemic antibiotics. We now have an opportunity to improve clinical precision of treatment and potentially expand prevention and treatment efforts. Clinicians should be able to improve antibiotic stewardship, reducing the use of antibiotic to the minimum necessary exposure. In addition, post-antibiotic therapies may be implemented to assist the body with restoration of a healthy microbiota. It is possible that future efforts may include non-antibiotic therapies aimed at optimizing the FUM—for treatment, as well as prevention, of certain urinary disorders.

As our knowledge of the FUM expands, there are research opportunities to investigate the etiology for a wide spectrum of poorly understood lower urinary tract disorders, including simple and recurrent UTI, overactive bladder syndrome, and UUI. It is possible, and indeed likely, that previously unrecognized microbes may play pathogenic roles alone or in combination with other members of the FUM, while others have protective, preventative roles.

Urgency urinary incontinence is a common disorder, affecting many adult women who experience bothersome urinary urgency, frequency, and urgency incontinence. Symptoms of UUI are highly variable within individuals and among affected women. Many affected women have persistent symptoms, despite some treatment efficacy from behavioral techniques, oral medications, and a variety of other modalities. Research into FUM shows an association between UUI symptom severity and certain FUM characteristics, including the number and variety of microbes detected. In addition, there are statistical associations between UUI symptoms and several bacterial species, including *Actinomyces neuii*, *Actinotignum schaalii* (formerly *Actinobaculum schaalii*), *Aerococcus urinae*, *Corynebacterium coyleae*, *Corynebacterium riegelii*, *Oligella urethralis*, and *Streptococcus anginosus*.^21^ Several of these species are considered uropathogens; however, most would not be detected by standard urine culture, high-lighting the need for the improved urine culture techniques described earlier in this paper. In a clinically significant study, researchers have detected differences in certain FUM characteristics of pre-treatment urine samples that appear to predict response to treatment with UUI medication. There was less microbial diversity in women who responded to oral medication UUI treatment than in those who did not or who required increased medication doses.^49^ These early studies suggest that UUI is heterogeneous and can be further phenotyped by FUM characteristics. This may allow a more personalized approach to UUI treatment, perhaps modulating FUM characteristics for a more favorable treatment response.

Another clinical area of importance for FUM research is the relationship with UTI. Readers are aware that the prior clinical concept of microbes in the female urinary bladder was dichotomous and limited to “sterile” versus “infected.” Because this dichotomy was not absolute, a third category was invented and termed “asymptomatic bacteriuria” to describe the clinical situation whereby a patient who does not have typical UTI symptoms has a positive standard urine culture. The therapeutic goal for UTI treatment is killing microbes with antibiotics. To date, we have not harnessed the innate human ability to prevent or fight uropathogens. For example, modulation of the urinary microbiota has not been utilized, and there is limited use of bacteriotherapy (such as microbial transplant).

Simple UTIs are common, relatively easily diagnosed, and often empirically treated. Treatment is typically based on eradication of a single uropathogen that has invaded the otherwise “sterile” bladder. However, in urogynecologic populations, clinicians also may find the simple diagnosis of UTI challenging, given the coexistence of frequency and urgency, chronic conditions for many urogynecologic patients.

Very little has been done to reduce the risk of UTI, especially during high-risk events such as genitourinary surgery, during which an estimated 20%–40% of patients experience at least one post-operative UTI. To date, research has confirmed that it is possible to identify a group at increased risk of UTI following surgery^53^ and reduce that risk with non-antibiotic therapy.^54^ These initial efforts highlight the possibility of dramatic reductions in surgery-associated UTI.

An important subset of women suffers from recurrent UTI. These patients experience life disruption from the frequent intrusion of symptomatic infection and repetitive courses of systemic antibiotics and/or low-dose antibiotic regimens for prevention of UTI recurrence.^55^ Researchers have learned that hormonal status appears related to FUM diversity^50^ and that an estrogenized genitourinary environment is likely beneficial for UTI reduction.^56^ Other results suggest that predominance (most often by *Lactobacillus* species) is typical of the bladder microbiota of hormone-positive women.^50^ This association is consistent with the clinically useful role estrogen has in suppressing recurrent UTI in certain hypoestrogenic women.^57^

Clinicians also may treat patients with various lower urinary tract symptoms of unclear etiology, urinary urgency, frequency, and/or bladder pain. Often, however, the usual work-up, including the standard urine culture, is negative. Many clinicians believe that the spectrum of bladder pain conditions has an “infectious” etiology, although previous culture-based microbial detection methods have not confirmed this suspicion. The long-held suspicion may be correct, as recent studies, using DNA-based approaches and voided urine samples, suggest that there may be a microbial component to these symptoms.^58^,^59^ Confirmation from on-going studies is underway using catheterized samples, in order to distinguish vulvo-vaginal contaminants from true members of the resident bladder microbiota. Until the results of these studies are available, affected patients and their clinicians may benefit from the use of enhanced urine culture techniques, described earlier in this paper.^28^ The results of these improved studies may help identify microbes not generally recognized as uropathogens; in select patients, these organisms may contribute to symptoms. For example, see Prigent et al. and Lotte et al.^60^,^61^

## FUM RESEARCH PRIORITIES

Now that we know that the FUM exists, what should clinicians expect? First and foremost, we must determine whether EQUC outperforms the standard urine culture protocol in terms of patient outcomes. Evidence that the standard urine culture misses 90% of the microbes detected by EQUC,^21^,^25^,^49^ and 50% of known or suspected uropathogens,^28^ suggests that diagnosis by EQUC will improve out-comes. Clinical trials should be designed and executed. During their design, clinicians and clinical microbiologists must take into account the lack of therapeutic algorithms and/or antibiotic sensitivity profiles for several emerging pathogens detected by EQUC, especially Gram-positives. To avoid vulvo-vaginal contamination and thus facilitate data interpretation, the urine samples to be tested must be collected by transurethral catheter or suprapubic aspirate. The results of such trials certainly would include comparative patient outcomes, but also evidence of failed therapeutic strategies and/or the presence of microbes not considered to be uropathogens.

The FUM can be diverse. From more than 1,000 study participants, researchers have detected almost 300 different species in about 150 different genera. What are those bacteria doing? Which ones are beneficial and should be protected? Which ones are pathogenic? Do presently unknown pathogens cause disorders that currently have no etiology, such as UUI and painful bladder syndrome? Do some bacteria (e.g. *Gardnerella vaginalis*) facilitate colonization by uropathogens such as UPEC? Do others inhibit the growth of pathogens? How do beneficial and pathogenic bacteria interact with the urothelium and its innate immune system?

## CONCLUSIONS

Astute clinicians will be faced with new paradigms for common urinary disorders—these paradigms will include the role of the human microbiome and the urinary microbiota. Clinicians must learn about the female urinary microbiota in order to under-stand research advances, as well as changes in clinical care algorithms. Improved clinical urine culture techniques will better describe the urinary microbes present at the time of sampling. Clinicians are likely to move away from the simplistic therapeutic goal of “killing microbes with antibiotics”; evolving therapies to enhance the beneficial microbes in the urinary community may replace or augment the current limited role of antibiotics. Beyond selection of an immediate treatment, clinicians must determine the relevance of the additional microbes detected with improved techniques. Further research is needed to determine whether these microbes are relevant to clinical conditions of interest. Our goal is to restore health, including restoring healthy microbial communities in the urinary system.

At this time, clinical use of improved culture techniques that provide additional microbial information may be of particular relevance for women whose standard urine cultures show no growth and whose symptoms persist. Vague clinical laboratory reports that suggest the presence of “mixed urethral flora” on a catheterized specimen are not clinically useful and may be an indication for expanded quantitative testing, as described earlier in this paper. Further research will help refine treatment algorithms and prevention strategies.

Much additional work needs to be done as researchers work to clarify the roles of urinary microbial communities and the role of specific organisms in maintaining urinary health. With the knowledge that some urinary microbes are playing an undoubtedly “good,” protective role, clinicians need to be open to improvements to the traditional, simple approach of widespread antibiotic use to eradicate all bladder organisms.

Research provides insights and raises questions yet to be answered. As additional researchers become trained in urinary microbial research, we fully expect that we will be able to make important clinical impacts that enhance our ability to prevent, diagnose, and treat—in a more targeted manner. In addition we may have the opportunity to provide “peri-antibiotic” care to optimize the urinary microbial community before, during, or after clinically indicated antibiotic use. These studies will be augmented by important studies of the immune functions of the lower urinary tract, both in health and in disease. Many adult women will not develop lower urinary disorders—incontinence or infection. Given that, it is indeed startling that, in 2017, we still do not understand many basic functions of the lower urinary tract and how those systems stay healthy, preventing UTI and preserving urinary continence. Since all prior lower urinary research was done without knowledge of the female urinary microbiota research, much prior work may need to be repeated or reinterpreted with this new knowledge. We look forward to the opportunity to provide updated information in several years as this bur-geoning area of research continues to flourish.

## Abbreviations

BAP: blood agar plate
CFU/mL: colony-forming units per milliliter
EQUC: enhanced quantitative urine culture
FUM: female urinary microbiota
HMP: Human Microbiome Project
NIH: National Institutes of Health
rRNA: ribosomal RNA
UPEC: uropathogenic *E. coli*
UTI: urinary tract infection
UUI: urgency urinary incontinence
